# Enhanced culturing techniques for the mycobiont isolated from the lichen *Xanthoria parietina*

**DOI:** 10.1007/s11557-021-01707-7

**Published:** 2021-06-07

**Authors:** Gregor Pichler, Fabio Candotto Carniel, Lucia Muggia, Andreas Holzinger, Mauro Tretiach, Ilse Kranner

**Affiliations:** 1 Department of Botany, University of Innsbruck, Sternwartestraße 15, 6020 Innsbruck, Austria; 2 Department of Life Sciences, University of Trieste, Via Giorgieri 10, 34127 Trieste, Italy

**Keywords:** Culture, D-arabitol, D-glucose, D-mannitol, Image Analysis, Ribitol

## Abstract

Lichens and their isolated symbionts are potentially valuable resources for biotechnological approaches. Especially mycobiont cultures that produce secondary lichen products are receiving increasing attention, but lichen mycobionts are notoriously slow-growing organisms. Sufficient biomass production often represents a limiting factor for scientific and biotechnological investigations, requiring improvement of existing culturing techniques as well as methods for non-invasive assessment of growth. Here, the effects of pH and the supplement of growth media with either D-glucose or three different sugar alcohols that commonly occur in lichens, D-arabitol, D-mannitol and ribitol, on the growth of the axenically cultured mycobiont isolated from the lichen *Xanthoria parietina* were tested. Either D-glucose or different sugar alcohols were offered to the fungus at different concentrations, and cumulative growth and growth rates were assessed using two-dimensional image analysis over a period of 8 weeks. The mycobiont grew at a pH range from 4.0 to 7.0, whereas no growth was observed at higher pH values. Varying the carbon source in Lilly-Barnett medium (LBM) by replacing 1% D-glucose used in the originally described LBM by either 1%, 2% or 3% of D-mannitol, or 3% of D-glucose increased fungal biomass production by up to 26%, with an exponential growth phase between 2 and 6 weeks after inoculation. In summary, we present protocols for enhanced culture conditions and non-invasive assessment of growth of axenically cultured lichen mycobionts using image analysis, which may be useful for scientific and biotechnological approaches requiring cultured lichen mycobionts.

**Supplementary Information:**

The online version contains supplementary material available at 10.1007/s11557-021-01707-7.

## Introduction

Lichens represent one of the most successful symbiotic associations on Earth and are capable of surviving in extreme environments (Kranner et al. 2008; Grube 2010; Meeßen et al. 2013a, 2013b), where life faces its limits (de Vera et al. 2008; de la Torre et al. 2010). Lichens comprise a fungal partner, the “mycobiont”, associated with at least one or more photoautotrophic partner(s), the “photobiont”, mostly green algae (“chlorobionts”), and/or cyanobacteria (“cyanobionts”) (Honegger 1991; Sanders 2001; Henskens et al. 2012). Lichens are also inhabited by bacteria and other microfungi (Grube and Berg 2009; Spribille et al. 2016; Muggia and Grube 2018; Hawksworth and Grube 2020). The fungal partner, which gives the name to the lichen, is responsible for building the complex structure of the lichen thallus, although it can only achieve this in symbiosis with a compatible photobiont (Honegger 1993; Kranner et al. 2005; Meeßen et al. 2013a).

Lichen mycobionts produce a plethora of secondary fungal products with antibiotic, antimycotic or antiviral properties (Halama and Van Haluwin 2004; Shresta and Clair 2013; Odimegwu et al. 2019), some of which may be of pharmaceutical interest (Müller 2001). For example, the anthraquinone parietin (=physcion) produced by *Xanthoria parietina* appears to have antioxidative, anti-bacterial, anti-tumour and laxative properties (Solhaug and Gauslaa 1996; Pang et al. 2016; Li et al. 2019). Considering the great potential of secondary lichen metabolites produced by lichen mycobionts (Calcott et al. 2018), biotechnological applications would benefit from efficient in vitro culturing techniques. However, lichen mycobionts — whether in symbiosis (Armstrong 1983; Honegger 1993) or in vitro (Ahmadjian 1961; Honegger et al. 1993, 1996; Pichler et al. 2020b) — are notoriously slow-growing organisms and many are endangered (Nascimbene et al. 2013), which often represents a limiting factor for scientific investigations and industrial applications.

Methods for the isolation of mycobionts from lichen thalli have been published since the 1960s (Ahmadjian 1961; Richardson and Smith 1968; see Yoshimura et al. 2002 for standard protocols). To maintain mycobiont growth in axenic culture, it is recommended to mechanically disrupt the fungal biomass regularly (Armaleo 1991) and to choose a growth medium containing appropriate amounts of carbon and nitrogen sources (Stocker-Wörgötter 2001). Growth media suitable to culture isolated mycobionts include undefined growth media that contain a complex mix of unknown chemical compounds, such as *Trebouxia* medium (TM; Ahmadjian 1987) and Malt-Yeast extract medium (MY), or defined growth media, whose chemical composition is known, such as Lilly-Barnett medium (LBM; Yoshimura et al. 2002; Muggia et al. 2017), and growing isolated lichen mycobionts on different growth media can result in remarkable phenotypic differences (Fazio et al. 2009).

Sugar alcohols may play pivotal roles in the lichen symbiosis (Palmqvist 2000; see Eisenreich et al. 2011 for a detailed review of sugar alcohol metabolism of lichens). Honegger et al. (1993) cultured eleven isolated lichen mycobionts, supplementing the growth media with glucose and maltose, to enhance biomass production, and detected species-specific variations in the sugar alcohols mannitol, arabitol, glycerol, volemitol and erythritol in mycobiont hyphae, of which mannitol and arabitol were detected in all mycobionts tested (Honegger et al. 1993). Therefore, mycobionts are apparently able to convert sugars into sugar alcohols. Komiya and Shibata (1971) found for two *Ramalina* species that ribitol, produced by the photobiont, was transported to the mycobiont and converted into arabitol and mannitol, whereas Wang et al. (2009) showed that ribitol significantly enhanced growth of several isolated mycobionts. Mannitol and ribitol also supported growth of lichenicolous fungi (Yoshino et al. 2020). Furthermore, ribitol and mannitol are known cryoprotectants, enhancing the solubilization of fungal enzymes in lichen thalli during freezing events (Fontaniella et al. 2000; Hájek et al. 2009a). In addition, ribitol induced a concentration-dependent increase in the maximum quantum yield of photosystem II (*F*_v_/*F*_m_) in lichen photobionts (Hájek et al. 2009b). Arabitol and ribitol were also identified as signalling compounds in lichens. Kosugi et al. (2013) observed in the lichen *Ramalina yasudae* and its isolated symbionts that ribitol produced by the photobiont was transferred to the mycobiont, converted to arabitol, and then transported back to the photobiont. They also showed that arabitol improved the ability to dissipate excess light energy when the photobiont was exposed to desiccation. It was possible to mimic this effect using D-arabitol, but not with its diastereomer L-arabitol or other sugar alcohols tested, such as mannitol, ribitol, sorbitol or xylitol. However, with the exception of the few above-mentioned reports, hardly any studies exist on the effects of sugar alcohols produced by lichen photobionts on mycobiont growth in culture.

In addition to nutrient composition, substrate pH also represents a crucial factor that affects developmental processes of lichens in their natural habitats as well as in isolated myco- and photobionts grown in in vitro culture (Herk 2001; Bačkor and Fahselt 2003; Bačkor et al. 2007). For example, species composition of epiphytic lichens in nature was shown to depend strongly on the pH of the tree bark they grew on (Herk 2001). Furthermore, most isolated mycobiont cultures only grew sufficiently in culture at a narrow pH range, often slightly acidic (Yoshimura et al. 2002). In addition, Timsina et al. (2013) found that biochemical processes, such as polyketide synthase gene activity, in the lichen-forming fungus *Ramalina dilacerata* is strongly affected by growth medium and pH, and Hamada (1989) observed that maximum depside production by the *Ramalina siliquosa* mycobiont occurred at the pH optimum for growth. In summary, growth and production of secondary lichen metabolites by mycobionts also depend on substrate pH.

The main goal of this study was to significantly improve biomass production of one representative isolated mycobiont and to accurately describe its growth phases in culture. We chose to work with the isolated mycobiont of the globally distributed lichen *Xanthoria parietina* (Honegger et al. 2004), an emerging model lichen (Itten and Honegger 2010) in the class Lecanoromycetes (Scherrer et al. 2005). In nature, *X. parietina* grows relatively fast (Honegger et al. 1996; Fortuna and Tretiach 2018), developing a foliose thallus on solid underground, e.g. bark or rocks (Lindblom and Ekman 2007; Beck and Mayr 2012). This lichen species is more resistant to environmental pollution than most other lichens (Armstrong and Bradwell 2010, 2011; Bertuzzi et al. 2018; Cecconi et al. 2019) and occurs also in urban, industrial and agricultural areas (Olsen et al. 2010; Vitali et al. 2019). We first identified the optimal pH range for culturing the *X. parietina* mycobiont on solid LBM and then studied the effects of D-glucose and the sugar alcohols D-arabitol, D-mannitol and ribitol on mycobiont growth, which was non-invasively monitored using two-dimensional image analysis.

## Materials and methods

### Strain identity and culture conditions

The axenic mycobiont *Xanthoria parietina* (L.) Th. Fr., (strain L 2379), grown from a single-spore isolate of the lichen *X. parietina*, was retrieved from the culture collection of the University of Trieste. The identity of the mycobiont was confirmed by ITS sequencing (ITS1, 5.8S, ITS2) and the corresponding NCBI GenBank accession number is MT513231, as described by Pichler et al. (2020b). Fungal stock cultures were grown in 50 mL of modified liquid Lilly-Barnett medium (LBM, pH 5.0) supplemented with additional 20 g L^−1^ of sucrose according to Pichler et al. (2020b). Cultures were kept in a growth chamber without shaking (Percival PGC-6HO, CLF Plant Climatics GmbH, Wertingen, Germany) under controlled conditions at 20 °C, 14/10 h light/dark regime and 20 μmol photons m^−2^ s^−1^, subsequently described as dim light (Pichler et al. 2020a, 2020b). To produce sufficient fungal biomass, the liquid LBM was renewed every 4 weeks and biomass was homogenized every 3 months (Yoshimura et al. 2002), in 2-mL Eppendorf tubes equipped with a steel grinding ball (3 mm in diameter) using a tissue-lyser (TissueLyser II, Qiagen, Düsseldorf, Germany) at a frequency of 30 Hz for 2 min. Then, new liquid cultures were re-started by re-inoculating homogenized fungal biomass in 50 mL of freshly prepared liquid LBM (pH 5.0 prior to autoclaving) and incubating as described above. All equipment was autoclaved and/or surface-sterilized before use.

### Inoculation method

Fungal cultures were inoculated following the methods described in Pichler et al. (2020b). 2 mL of fungal liquid culture, grown as described above, was transferred to 2-mL Eppendorf tubes (12 tubes in total), centrifuged at 800×*g* at 15 °C for 2 min (Sigma® 3-18 KS) and the supernatants were discarded. To remove the liquid growth medium from the fungal biomass, 1 mL of distilled water (dH_2_O) was added, followed by vortexing for 5 s and centrifugation (800×*g* and 15 °C for 2 min). Then, the supernatant was removed again, another 1 mL dH_2_O and a steel ball (5 mm in diameter; pre-cleaned with acetone and autoclaved) were added to each tube and the fungal biomass was homogenized with a tissue-lyser at 30 Hz for 2 min until a homogenous fungal suspension was obtained. The fungal suspensions of the 12 tubes were pooled in a 50-mL Erlenmeyer flask, and 500 μL were filtered through a hydrophilic polytetrafluoroethylene (PTFE) membrane (25 mm in diameter, pore size 0.45 μm, Omnipore™, Ireland) placed in a glass-metal filtration system (Sartorius-Membranfilter GmbH, Göttingen, Germany) with a manual vacuum pump (MV8529, Mitycac®, St. Louis, USA). This filtration step was conducted three times and the filters supporting the fungal biomass were dried in the oven at 80 °C for 3 h until dry mass (DM) was stable. The mean DM of three such filters was used to calculate the fungal DM concentration in mg mL^-1^ suspension. Then, the fungal suspension was adjusted to a final concentration of 2 mg fungal DM mL^−1^ by adding dH_2_O. Three replicates of 100 μL of each fungal suspension were examined with a microscope (Zeiss Axiovert 200 M, Jena, Germany) to assure that fungal cell structures were intact, and photos were taken with a digital camera (Zeiss AxioCam HRc, Jena, Germany).

Then, 500 μL of fungal suspension was inoculated on hydrophilic PTFE filters (25 mm in diameter, mesh size 0.45 μm) and filtered using a glass-metal filtration system (as above) with a manual vacuum pump to remove extracellular fluids, leaving only fungal biomass on the PTFE filter. Each PTFE filter supporting the fungal biomass was placed onto solid LBM (2% agar) in Petri dishes (55 mm in diameter, polystyrol (PS) Petri dishes, Rotilabo®, Germany) at either different pH values or supplemented either with D-glucose or different sugar alcohols (see below).

### pH-dependent growth of the *X. parietina* mycobiont

The optimal pH range to culture the mycobiont of *X. parietina* was assessed by adjusting the solid LBM (2% agar) to pH values of 4.0, 5.0, 6.0, 7.0, 8.0, 9.0, 10.0, 11.0 and 12.0, with 10 M, 1 M and/or 0.1 M solutions of HCl or NaOH prior to autoclaving. L-asparagine, D-glucose and the vitamins biotin and thiamine were dissolved in dH_2_O and the pH was adjusted separately prior to sterile filtration, and then these components were added to the autoclaved growth medium when the temperature had decreased to 55 °C. The original pH values of 4.0, 5.0, 6.0, 7.0, 8.0 and 9.0 were confirmed to remain stable in the medium (with a deviation of ± 0.1) after autoclaving and solidification, whereas the initial pH values of 10.0, 11.0 and 12.0 slightly decreased to 9.5 ± 0.0, 10.1 ± 0.0 and 11.5 ± 0.0, respectively (Table S1). Preparation of solid LBM with a pH lower than 4.0 or higher than 12.0 prior to autoclaving failed, as the medium did not solidify.

PTFE filters were inoculated with the mycobiont suspension, as described above, and transferred onto solid standard LBM (2% agar) of different pH values. After 8 weeks of growth in dim light, fungal biomass was harvested, transferred to 2-mL Eppendorf tubes and freeze-dried for 90 h, according to Bailly and Kranner (2011). The DM of each biological replicate (*n* = 6) was measured with an analytical balance (XS 105, ©Mettler Toledo, Austria).

### Sugar- and sugar alcohol–dependent growth of the *X. parietina* mycobiont

PTFE filters were inoculated with fungal biomass (corresponding to 1 mg DM, as described above) and then transferred to solid LBM with a pH of 6.0 (*n*=6 biological replicates). Standard LBM (see Yoshimura et al. 2002) containing 1% of D-glucose was used as a control to be compared with LBM supplemented with different concentrations of D-glucose or sugar alcohols as follows: instead of using 1% of D-glucose, LBM was supplemented with either D-glucose at concentrations of 2% or 3%, or with D-arabitol, D-mannitol or ribitol at concentrations of 1%, 2% or 3%. Concentrations of glucose or sugar alcohols higher than 3% were not used, because this concentration represented the limit to supplement the growth medium with sugar or sugar alcohols without immediate solidification. Mycobiont cultures were grown for 8 weeks under dim light, as described above. Then, each filter was transferred onto the glass-metal filtration system, and fungal biomass was washed with 500 μL of dH_2_O to avoid the different masses of D-glucose or sugar alcohols with which the LBM was supplemented, confounding the measurements of fungal DM. Then, the washing solution was removed using a manual vacuum pump. Fungal biomass was harvested and freeze-dried and DM was determined as described above.

### Assessment of cumulative growth and growth rate

**Fig. 1:**
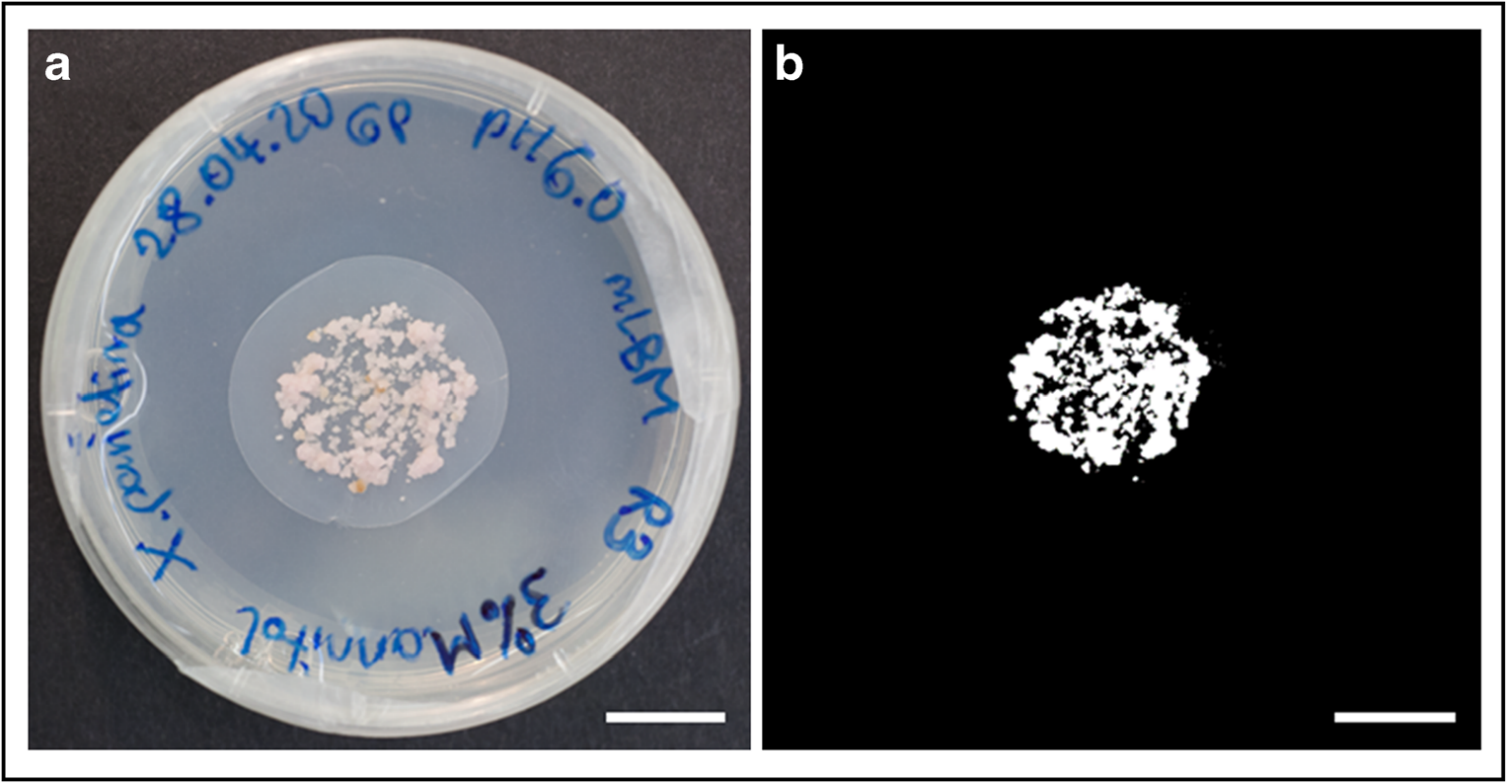
Image analysis to measure the area covered by the fungus. The area covered by the mycelium was measured to determine growth of the *Xanthoria parietina* mycobiont over eight weeks. **a** Digital image of a mycobiont culture; **b** conversion of the image shown in panel **a** to an 8-bit black and white image with the fungal area in the centre in false colours (white); scale bar = 1 cm

Mycobiont cultures grown on LBM supplemented with 3% of either D-arabitol, D-glucose, D-mannitol or ribitol instead of 1% D-glucose were photographed every second week for up to 8 weeks. Photos were taken with a digital full-frame camera (EOS 5D, Canon Inc., Japan) connected via a lens mount adapter (Shenzhen Neewer Technology Co., Guangdong, China) to a manual macro lens set at aperture f5.6 (Zuiko MC Auto-Macro 1:3.5/50 mm, Olympus®, Japan). White balance of .CR2 raw files was adapted and files were converted to .tiff format with Adobe Photoshop CS6 (Version 13.0 20120315.r.428) prior to image analysis with ImageJ (version 1.53c; Rehorska et al. 2014). The image analysis method described by Ametrano et al. (2017) was used with some modifications, as follows. Each photo was converted to 8-bit black and white format using the “type” function. After setting a precise scale, the clearly visible area of fungal hyphae was marked using the “threshold” function and the covered area was measured and expressed in square centimetres (Fig. 1). Cumulative growth was defined as total area covered by fungal hyphae (cm^2^) over time at weeks 0, 2, 4, 6 and 8. Growth rate was defined as the increase in area newly covered by fungal hyphae every week (cm^2^ week^−1^) and calculated for each of the time points.

### Statistics

The software R (Version 3.5.1) and RStudio (Version 1.1.383) were used for statistical analyses. Normal distribution of data was tested with QQ-plots and the Shapiro-Wilk test. A non-parametric Kruskal-Wallis test (*p*-value < 0.05), followed by Dunn’s post hoc test (*p*-value < 0.05) with Benjamini-Hochberg correction, was conducted to assess significant differences (*p*-value < 0.05) between (i) DMs grown on LBM with different pH and (ii) cumulative growth data of *X. parietina* grown with D-arabitol, D-glucose, D-mannitol and ribitol at a concentration of 3% at week 8. For each time point, significant differences (*p*-value < 0.05) between growth rates were assessed by the non-parametric two-sided Mann-Whitney *U* Tests. For multiple parameter testing, non-parametric two-sided Mann-Whitney *U* tests (*p*-value < 0.05) with Benjamini-Hochberg correction was used to identify significant differences (*p*-value < 0.05) between DMs of cultures grown on LBM supplemented with either D-arabitol, D-glucose, D-mannitol or ribitol, each at concentrations of 1%, 2% and 3%.

## Results

### Effects of pH on growth of the *X. parietina* mycobiont

**Fig. 2:**
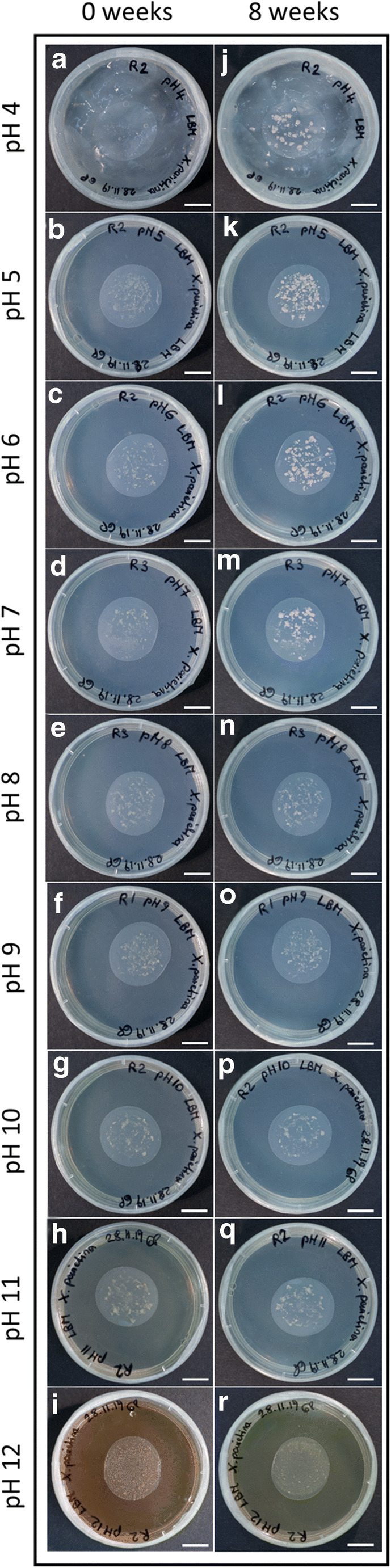
Growth of the *Xanthoria parietina* mycobiont on solid Lilly-Barnett medium with pH values ranging from 4 to 12: fungal cultures (**a**−**i**) 4 days after inoculation, termed as "0 weeks", and (**j**−**r**) after 8 weeks ; scale bar = 1 cm

**Fig. 3:**
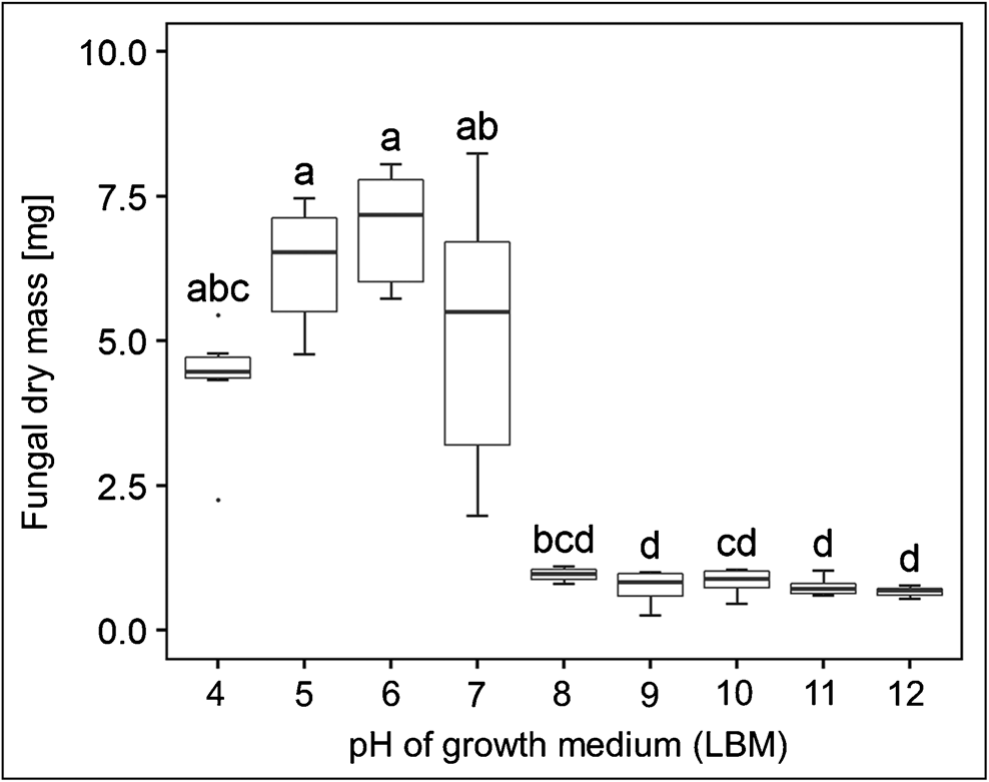
Effects of pH on growth of the mycobiont *Xanthoria parietina*. Dry mass is shown for fungal cultures grown for 8 weeks on solid Lilly-Barnett medium at different pH values. Boxplots show median, 25th and 75th percentiles, maxima and minima, and outliers (dots); *n* = 6 biological replicates. Statistically significant differences, assessed with the Kruskal-Wallis test (*p*-value < 0.05), are indicated by different letters above the boxplots

The mycobiont of *X. parietina* was able to grow on solid standard LBM with a pH ranging from 4.0 to 7.0 (Fig. 2j–m and Fig. 3), whereas no growth was observed on media with a pH higher than 7.0 (Fig. 2n–r and Fig. 3). DMs produced in the range of pH 4.0 to 7.0 did not significantly differ (*p*-value < 0.05) from each other, and as growth tended to be best at pH 6.0, this was selected for further experiments.

### Effects of different concentrations of D-glucose and sugar alcohols on biomass production of the *X. parietina* mycobiont

**Fig. 4:**
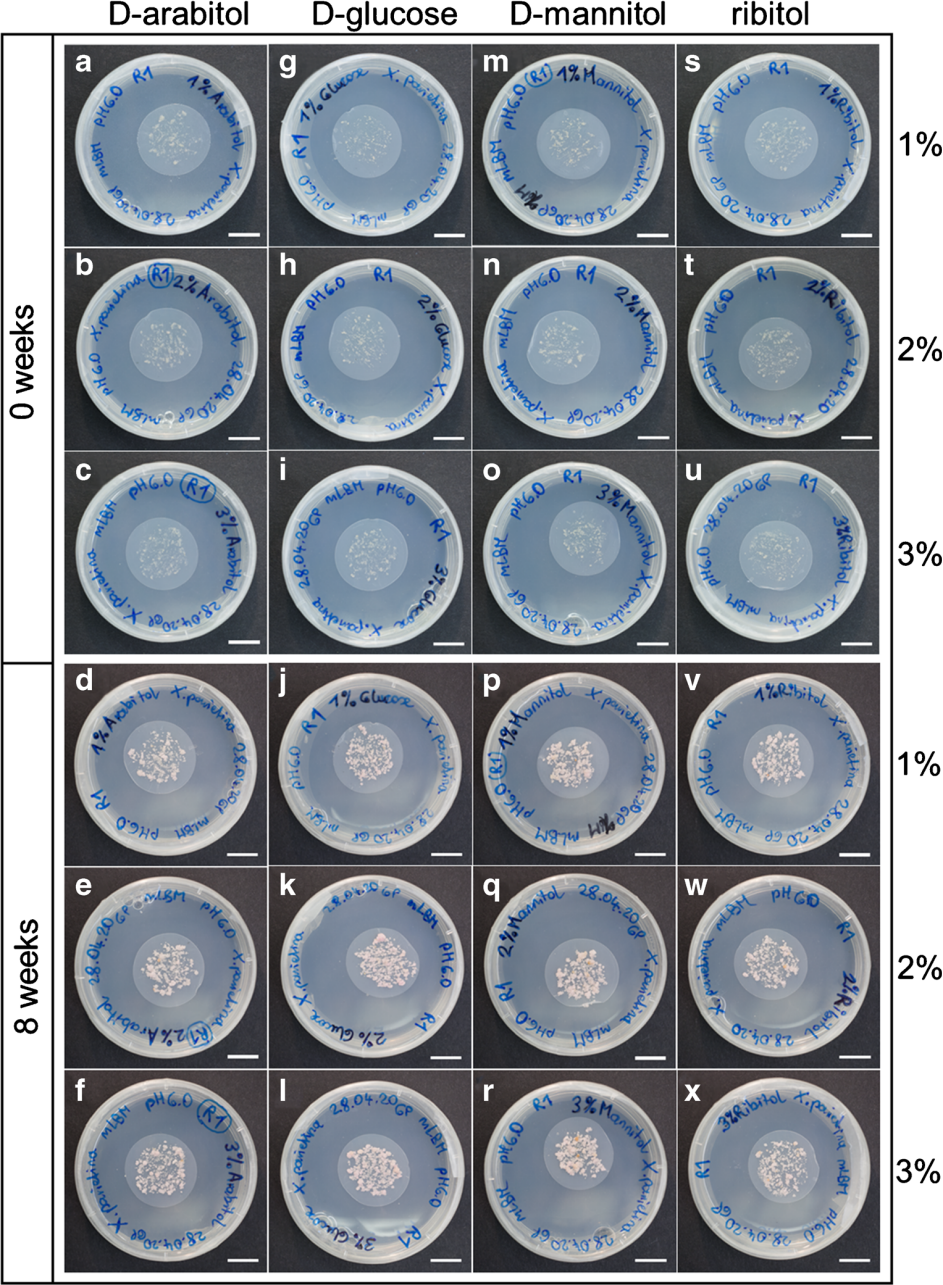
Effect of D-glucose and sugar alcohols on growth of the *Xanthoria parietina* mycobiont*.* Cultures were grown on solid Lilly-Barnett medium containing **a**–**f** D-arabitol, **g**–**l** D-glucose, **m**–**r** D-mannitol and **s–x** ribitol, each at concentrations of 1%, 2% and 3%; the upper half of the figure shows photos of fungal cultures taken 4 days after inoculation and the lower half of the figure shows fungal cultures 8 weeks after inoculation; scale bar = 1 cm

**Fig. 5:**
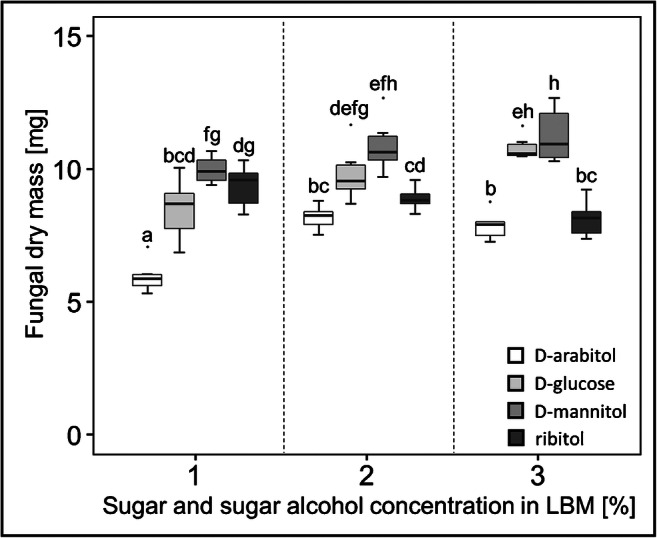
Growth of the *Xanthoria parietina* mycobiont at different sugar and sugar alcohol concentrations, assessed by dry mass. The mycobiont was grown on solid Lilly-Barnett medium (LBM) containing either D-arabitol (white), D-glucose (light grey), D-mannitol (grey) or ribitol (dark grey) at concentrations of 1%, 2% and 3%, whereby the standard LBM medium containing 1% D-glucose was regarded as a control. Boxplots show median, 25th and 75th percentiles with maxima, minima and outliers (dots); *n* = 6 biological replicates. Statistically significant differences, assessed by multiple Mann-Whitney *U* tests, are indicated by different letters above the boxplots

The mycobiont was able to grow on LBM supplemented with either D-arabitol, D-glucose, D-mannitol or ribitol at concentrations of 1%, 2% and 3% (Fig. 4). Compared to standard LBM containing 1% of D-glucose, fungal DM significantly increased (*p*-value < 0.05) by 14%, 23%, 26% and 22%, when 1%, 2% and 3% of D-mannitol or 3% D-glucose, were present in the growth medium, respectively (Fig. 5). Furthermore, increasing concentrations of D-arabitol, D-glucose, D-mannitol and ribitol from 1 to 3% affected the growth of *X. parietina* differently, depending on the sugar or sugar alcohol used. Compared to the 1% concentrations, fungal growth was significantly enhanced (*p*-value < 0.05) by higher concentrations of D-arabitol (2% and 3%), D-glucose (3%) and D-mannitol (3%). In contrast, fungal biomass production decreased when 3% ribitol was present (Fig. 5). No significant differences (*p*-value < 0.05) in DM between 2 and 3% D-arabitol were observed. In summary, when offered at concentrations of 1%, D-mannitol supported fungal growth more than D-glucose, D-arabitol or ribitol, and fungal biomass increased the most when D-mannitol at concentrations of 2% and 3%, or D-glucose at 3%, were offered.

### Effects of 3% D-glucose or sugar alcohols on cumulative growth and growth rate of the *X. parietina* mycobiont

**Fig. 6:**
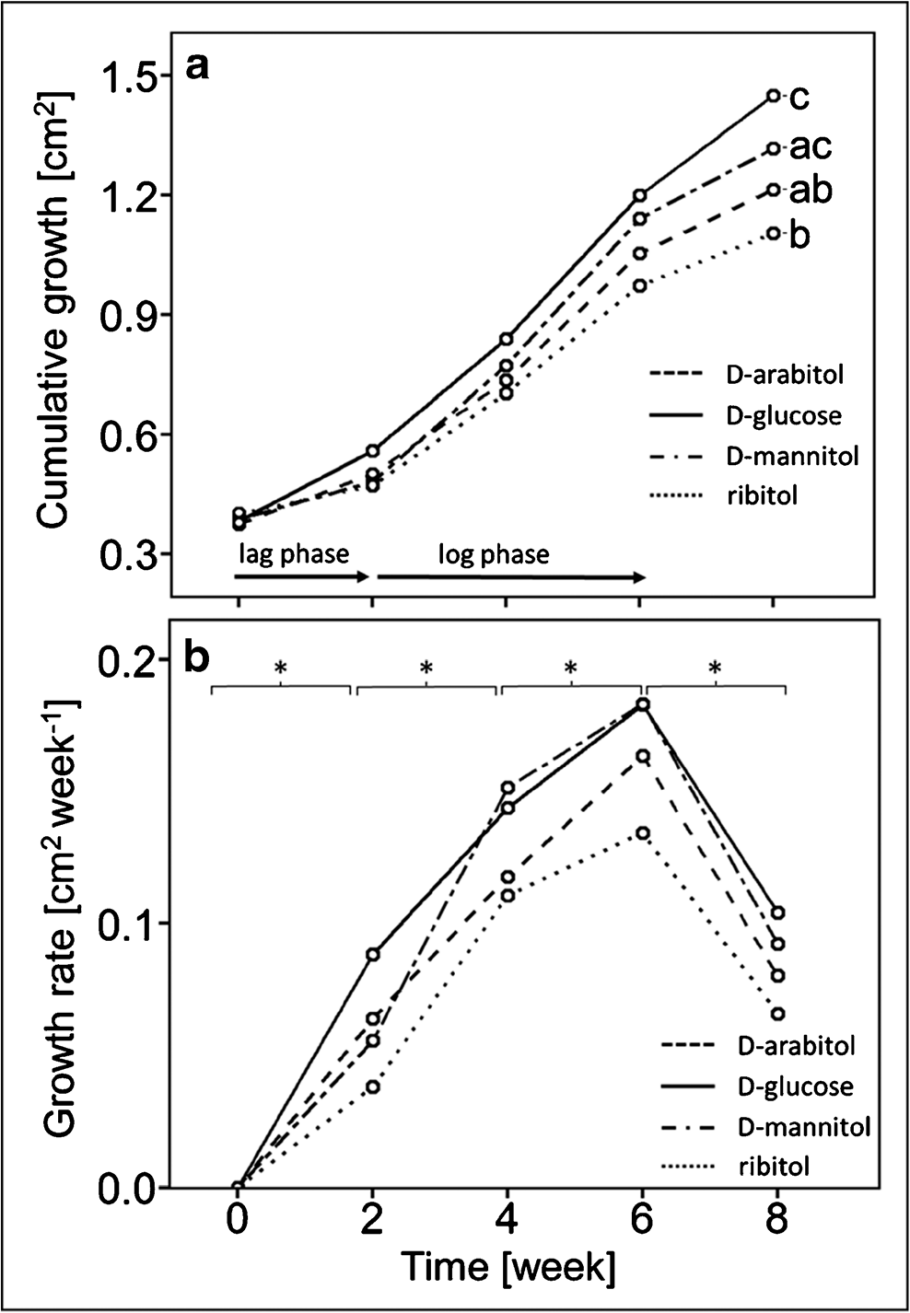
Effects of D-glucose and sugar alcohols on growth of the *Xanthoria parietina* mycobiont. Cultures were grown for 8 weeks on solid Lilly-Barnett medium, containing 3% of either D-arabitol, D-glucose, D-mannitol or ribitol, indicated by dashed, solid, two-dashed and dotted lines, respectively. **a** Cumulative growth was assessed by the total fungal area (cm^2^); dots represent median values. The arrows show lag and log phases; statistically significant differences, assessed by the Kruskal-Wallis test (*p*-value < 0.05) are marked by different letters; *n*= 6 biological replicates. **b** Growth rates, assessed by the change in area covered by the fungus per week (cm^2^ week^−1^); dots represent median values; statistically significant differences between time intervals for each treatment, assessed with the Mann-Whitney *U* test (*p*-value < 0.05) are marked with asterisks; *n* = 6 biological replicates

As most pronounced differences between media supplemented with 3% D-glucose or sugar alcohols on mycobiont growth were observed, with D-glucose and D-mannitol resulting in highest- and ribitol and D-arabitol resulting in lowest growth (Fig. 5), we further assessed growth phases at this concentration. Photos of fungal cultures grown on LBM supplemented with 3% of either D-arabitol, D-glucose, D-mannitol or ribitol were analysed by ImageJ to assess cumulative growth and growth rate over 8 weeks. The cumulative growth of the *X. parietina* mycobiont treated with 3% of either D-arabitol, D-glucose, D-mannitol or ribitol increased in a sigmoidal manner, with an initial lag phase until week 2, followed by a log phase with exponential growth between weeks 2 and 6 (Fig. 6a; Table S2). After 8 weeks, the total area covered by the fungus was largest after treatment with 3% D-glucose and D-mannitol and smallest for 3% ribitol (Fig. 6a), in agreement with data shown in Fig. 5. Growth rates significantly (*p*-value < 0.05) increased between inoculation and week 6, then significantly (*p*-value < 0.05) decreased between weeks 6 and 8 (Fig. 6b). Growth rate curves (Fig. 6b) fitted with polynomial functions of degree 3 (for more details, see Table S2).

## Discussion

Secondary lichen metabolites primarily produced by mycobionts, such as the anthraquinone parietin (also termed physcion), parietinic acid, emodin, fallacinal or teloschistin produced by *X. parietina*, receive increasing attention due to their potential medicinal properties (Boustie and Grube 2005; Basile et al. 2015; Łaska et al. 2016). However, the use of mycobiont cultures for producing metabolites of interest is often compromised by the slow growth of isolated mycobionts as well as their requirement of being co-cultured with their compatible photobionts, without which they do not produce the same set of secondary metabolites and often only low amounts thereof (Leuckert et al. 1990; Elshobary et al. 2016; Calcott et al. 2018). However, Culberson and Armaleo (1992) showed that production of secondary lichen metabolites by axenically grown mycobionts can be stimulated by decreasing the water contents of the mycobionts. Therefore, it is likely that solid rather than liquid growth media support the production of secondary lichen metabolites, in agreement with the occurrence of lichens in terrestrial environments (Asplund and Wardle 2017). Here, we focussed on optimizing biomass production of the *X. parietina* mycobiont for small-scale scientific applications, by modifying pH and carbon source in the growth medium, and show that image analysis is suitable to non-invasively assess mycobiont growth.

The pH of the growth medium is a crucial factor that affects growth of lichen mycobionts. Ahmadjian (1961) tested 18 isolated mycobionts and found that pH values between 4.5 and 6.5 were optimal for culturing, and a pH of 6 was used to successfully grow *X. parietina* in culture (Lenton et al. 1973; Honegger et al. 1993). Yoshimura et al. (2002) summarized that the optimal pH to grow lichen mycobionts is in the slightly acidic range for most species, often between pH 5 and 6. The optimal pH for mycobiont growth seems to be species-specific (Yoshimura et al. 2002) and likely related to the preferred ecological niche of the respective lichen (Glime and Iwatsuki 1990). *Xanthoria parietina* thrives on tree bark (Richardson 1967; Brunialti and Frati 2007), and the pH of the bark of four representative tree genera, *Quercus*, *Ulmus*, *Fraxinus* and *Tilia*, was reported to range from 4 to 7 (Spier et al. 2010), in agreement with our findings that the highest amounts of fungal biomass were produced when the *X. parietina* mycobiont was grown at pH values between 4 and 7 (Fig. 3). Hamada (1989) reported that the mycobiont isolated from *Ramalia siliquosa* grew well between pH 5 and 9, with an optimum at pH 6.5, but we did not observe growth of the *X. parietina* mycobiont above a pH of 7. The pH requirement for germination of ascospores from various lichen species also varied with species (Yamamoto et al. 1998). Ascospores of *X. parietina* were found to germinate at pH 5 to 7, with an optimum at pH 6 (Chrismas 1980), also in agreement with the pH range suitable for mycobiont growth.

After confirming the optimal pH range for culturing the *X. parietina* mycobiont, we studied the individual effects of D-glucose and three sugar alcohols on growth. The sugar alcohols D-arabitol, D-mannitol and ribitol are known to occur in lichens and their isolated symbionts (Komiya and Shibata 1971; Honegger et al. 1993; Alam et al. 2015) and to play important roles for the lichen symbiosis (Hájek et al. 2009a, 2009b; Kosugi et al. 2013). Ribitol and mannitol were also found in *X. parietina* symbionts, where these metabolites act as important energy source for metabolic processes (Eisenreich et al. 2011). However, their application to improve biomass production of isolated mycobionts has, to our knowledge, been barely tested (e.g. Wang et al. 2009; Alam et al. 2015). Therefore, we evaluated whether D-glucose, the standard carbon source in LBM, can be replaced by these sugar alcohols. Observed visually at a macroscopic level, no obvious differences in phenotype and secondary metabolite production were found in *X. parietina* mycobiont cultures depending on the sugar alcohol used, unlike described by Stocker-Wörgötter et al. (2009) for several *Haematomma* species. When LBM was supplemented with 1–3% D-glucose or sugar alcohols, a pale yellow- to orange-coloured biomass was produced in all cases (Fig. 4), indicative of the presence of parietin, a typical secondary metabolite of *X. parietina* (Solhaug and Gauslaa 1996). Furthermore, culturing the *X. parietina* mycobiont on modified LBM with 4% of glucose increased hyphal growth and diameter, when compared to nutrient-poor BBM (Molina and Crespo 2000). However, ascospores of *X. parietina* germinated best on BBM, although proper hyphal development required transfer to nutrient-rich medium (Molina et al. 1997; Molina and Crespo 2000). Testing the effects of increasing sucrose concentration (1, 2 and 3%) in solid MY medium on the mycobiont of *Evernia esorediosa*, Hamada et al. (1994) observed highest biomass production at concentrations of 1% and 2% sucrose, and concentrations of sugar and sugar alcohols produced by the mycobiont were influenced by the concentration of sucrose offered in the growth medium (Hamada et al. 1994). In the present study, the *X. parietina* mycobiont developed the highest amounts of biomass when cultured on either 3% D-glucose or 1%, 2% and 3% D-mannitol compared to standard LBM (Fig. 5). In addition, biomass production increased with increasing concentration of D-glucose, D-mannitol and D-arabitol in the LBM. Ribitol was observed to enhance growth of the mycobionts of *Ramalina farinacea* and *R. fastigiata* by up to 35.3% (Wang et al. 2009), but we observed that increasing ribitol concentrations decreased biomass production in *X. parietina* (Fig. 5). Others showed that growth of *Usnea longissima* and *U. diffracta* increased when cultured on LBM supplemented with mannitol at the concentrations of 2%, 4% and 6% and of 4% and 8%, respectively (Yamamoto et al. 1987). In a lichen thallus, ribitol is produced by the photobiont and transferred to the mycobiont, which then converts it into mannitol and/or arabitol (Komiya and Shibata 1971). Furthermore, Kosugi et al. (2013) found that D-arabitol may be transported back to the photobiont and suggested a putative role for D-arabitol in photo-protection. Mannitol, on the other hand, was incorporated into the cell walls of isolated mycobionts (e.g. *X*. *parietina*, *Tornabenia intricata* and *Sarcogyne* sp.), but not those of free-living fungi, assessed by the uptake of radioactively labelled [^3^H] mannitol (Galun et al. 1976). Mannitol is also an important storage compound required for morphogenesis and conidiation, and it may confer stress tolerance to free-living filamentous fungi (Solomon et al. 2007). Taken together with our observations that growth of *X. parietina* was greatly stimulated by D-mannitol at all three concentrations offered, when compared to standard LBM, it appears that D-mannitol is an excellent carbon source for lichen mycobionts. At 1 and 2% concentrations, D-mannitol was even better than D-glucose, so that we recommend considering supplementing mycobiont growth media with D-mannitol.

The growth of filamentous fungi in culture has been described to start with a lag phase, followed by a first transition phase, a log phase, a second transition phase and a stationary phase (Meletiadis et al. 2001). Using image analysis to non-invasively measure the total area covered by fungal hyphae over 8 weeks revealed a sigmoidal curve for cumulative growth, with exponential growth between week 2 and week 6 (Fig. 6a; Table S2). Growth rates increased significantly (*p*-value < 0.05) between inoculation and week 6, decreased significantly (*p*-value < 0.05) between weeks 6 and 8, and fitted with polynomial functions of degree 3 (Fig. 6b; Table S2). Ametrano et al. (2017) also used two-dimensional image analysis to assess growth rates of co-cultured dothidealean rock-inhabiting fungi and lichen photobionts on nutrient-poor and nutrient-rich media. To the best of our knowledge, no other studies are available that describe the different growth phases of lichen mycobionts. However, characterization of growth phases is important for standardization of experiments, and to produce robust and reproducible results, as described for free-living filamentous fungi (Meletiadis et al. 2001; Vrabl et al. 2019).

In summary, we showed that the optimal pH range to culture *X. parietina* is between pH 4.0 and 7.0, with an optimum around pH 6.0. Moreover, fungal biomass production can be significantly enhanced by all applied concentrations of D-mannitol and 3% D-glucose compared to the standard LBM, and at concentrations of 1 and 2%, D-mannitol supports growth even better than D-glucose. Therefore, sugar alcohols that occur in lichens and are transported between the symbionts, appear to be good alternatives to D-glucose for culturing of mycobionts. Thirdly, we showed that two-dimensional image analysis is a useful tool to non-invasively screen mycobiont growth on solid growth medium, which together with the use of suitable carbon sources may support future biotechnological uses of cultured lichen mycobionts.

## Supplementary Information

Table S1 (DOCX 23 kb)

Table S2 (DOCX 23 kb)

## Data Availability

All data generated or analysed during this study are included in this published article and its supplementary information files.

## Code availability

Data were generated using the software R (Version 3.5.1) and RStudio (Version 1.1.383).
