# Supplementary material for: Enhanced culturing techniques for the mycobiont isolated from the lichen Xanthoria parietina
Source: Mycol Prog. 2021 Jun 7;20(6):797–808. doi: 10.1007/s11557-021-01707-7 (PMC8550697; doi:10.1007/s11557-021-01707-7)
Supplement: Supplementary file 1 — (DOCX 23 kb) [file 11557_2021_1707_MOESM1_ESM.docx]

**Supplementary material**

**Table S1** pH values of solid Lilly-Barnett Medium during the experiment shown in Fig. 2 and Fig. 3. Data are either single values of the initial pH or mean ± SD after *i*) autoclaving, and *ii*) 8 weeks of growth of fungal cultures

| **pH of Lilly-Barnett Medium** | | |
| --- | --- | --- |
| initial  (n = 1) | after autoclaving  (n=3) | 8 weeks after inoculation  (n=6) |
| 4.00 | 3.97 ± 0.01 | 6.41 ± 0.22 |
| 5.00 | 4.94 ± 0.02 | 6.49 ± 0.12 |
| 6.00 | 5.91 ± 0.02 | 6.74 ± 0.04 |
| 7.00 | 6.95 ± 0.02 | 7.14 ± 0.05 |
| 8.00 | 7.90 ± 0.01 | 7.60 ± 0.01 |
| 9.00 | 8.90 ± 0.02 | 8.40 ± 0.01 |
| 10.00 | 9.46 ± 0.03 | 8.66 ± 0.02 |
| 11.00 | 10.06 ± 0.02 | 8.84 ± 0.03 |
| 12.00 | 11.50 ± 0.02 | 8.88 ± 0.02 |
