# Supplementary material for: Enhanced culturing techniques for the mycobiont isolated from the lichen Xanthoria parietina
Source: Mycol Prog. 2021 Jun 7;20(6):797–808. doi: 10.1007/s11557-021-01707-7 (PMC8550697; doi:10.1007/s11557-021-01707-7)
Supplement: Supplementary file 2 — (DOCX 23 kb) [file 11557_2021_1707_MOESM2_ESM.docx]

**Table S2** Mathematical description of growth curve kinetics of the *Xanthoria parietina* mycobiont. Equations, calculated from growth data displayed in Fig. 6, are shown, which describe functions of **a)** exponential cumulative growth in the log phase between week 2 to 6, and **b)** a polynomial growth rate between week 0 and 8, grown on Lilly-Barnett Medium, containing 3% of either D-glucose, D-arabitol, D-mannitol or ribitol; R-squared shows the coefficient of determination

| **a) Cumulative growth** | **(week 2 to 6)** |  |  |
| --- | --- | --- | --- |
| **Sugar/sugar alcohol** | **Equation** | **R^2^** | **Type of function** |
|  |  |  |  |
| D-glucose | y = 0.3853e^0.1907x^ | 0.9988 | exponential |
| D-mannitol | y = 0.3171e^0.2158x^ | 0.9969 | exponential |
| D-arabitol | y = 0.3458e^0.1864x^ | 0.9996 | exponential |
| ribitol | y = 0.334e^0.1802x^ | 0.9964 | exponential |
|  |  |  |  |
| **b) Growth rates** | **(week 0 to 8)** |  |  |
| **Sugar/sugar alcohol** | **Equation** | **R^2^** | **Type of function** |
|  |  |  |  |
| D-glucose | y = -0.0009x^3^ + 0.0041x^2^ + 0.036x | 0.9896 | polynomial^3^ |
| D-mannitol | y = -0.0017x^3^ + 0.0141x^2^ + 0.0079x | 0.9986 | polynomial^3^ |
| D-arabitol | y = -0.0012x^3^ + 0.0092x^2^ + 0.0149x | 0.9850 | polynomial^3^ |
| ribitol | y = -0.0013x^3^ + 0.0112x^2^ + 0.0031x | 0.9981 | polynomial^3^ |
|  |  |  |  |
